# Autologous Dermis Graft Implantation: A Novel Approach to Reinforcement in Giant Hiatal Hernias

**DOI:** 10.1155/2018/9069430

**Published:** 2018-05-08

**Authors:** Balázs Kovács, Mikolt Orosz, Máté Csucska, Saurabh Singhal, Árpád Juhász, Zoltán Lóderer

**Affiliations:** ^1^Department of Vascular, Plastic and General Surgery, Markusovszky University Teaching Hospital, Szombathely, Hungary; ^2^Norton Thoracic Institute, St. Joseph's Hospital and Medical Center, Phoenix, AZ, USA

## Abstract

**Objectives:**

Nonreinforced tensile repair of giant hiatal hernias is susceptible to recurrence, and the role of mesh graft implantation remains controversial. Creating a new and viable choice without the use of high-cost biological allografts is desirable. This study presents the application of dermis graft reinforcement, a cost-efficient, easily adaptable alternative, in graft reinforcement of giant hiatal hernia repairs.

**Methods:**

A 62-year-old female patient with recurrent giant hiatal hernia (9 × 11 cm) and upside down stomach, immediately following the Belsey repair done in another department, was selected for the pilot procedure. The standard three-stitch nonabsorbable reconstruction of diaphragmatic crura was undertaken via laparoscopic approach. A 12 × 6 cm dermis autograft was harvested from the loose abdominal skin. “U” figure onlay reinforcement of diaphragm closure was secured with titanium staples. The procedure was completed with a standard Dor fundoplication. One- and seven-month follow-ups were conducted.

**Results:**

No short-term postoperative complications were observed. One-month follow-up showed normal anatomical location of abdominal viscera on computed tomography imaging. High-resolution manometry showed normal lower esophageal sphincter pressure. Preoperative abdominal complaints were resolved. Procedural costs were lower than the average cost following mesh graft reinforcement.

**Conclusion:**

Dermis graft reinforcement is a cheap, easily adaptable procedure in the repair of giant hiatal hernias, even in the setting of laparoscopic reoperative procedure.

## 1. Introduction

**Figure 1:**
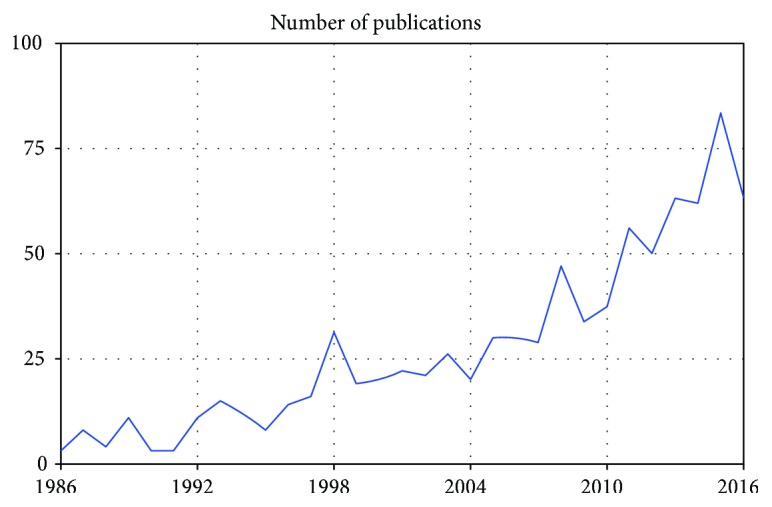
Number of publications per year on PubMed search for keywords “hiatal hernia repair.”

As the number of surgical interventions required for gastroesophageal reflux disease (GERD) and/or hiatal hernias is increasing worldwide, surgeons encounter increasingly more complicated and inveterate cases with large hiatal defects. The number of publications per year related to hiatal repairs has almost doubled in the last decade (Figure 1).

Pearson et al., in their classification of hiatal hernias in 1983, defined giant hiatal defect as the herniation of 30% or more of the stomach into the thoracic cavity in the posterior mediastinum [1]. Koch et al. later defined large hernias as the ones with the surface area of hiatal defect larger than 5 cm^2^ [2]. Laparoscopic repair of giant hiatal hernias presents a new challenge in the operative treatment. These defects are highly susceptible to recurrence, if repaired in a nonreinforced tensile manner [3]. The aim of this study was to find a feasible and safe technique to reinforce this hiatal gap.

## 2. Methods

**Figure 2:**
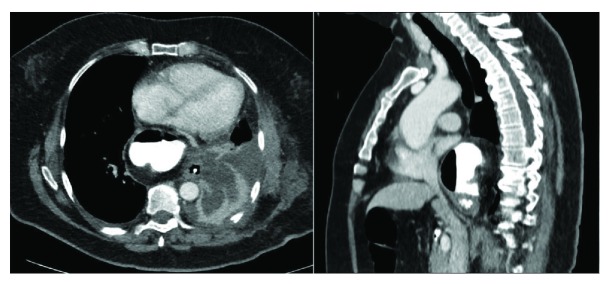
Preoperative CAT images show paraoesophageal herniation of the antrum into the thoracic cavity.

A 62-year-old female patient, with a history of complication during the reversal of anesthesia following varicectomy procedure, underwent repeated evaluations for complaints of dyspnoea on exertion and epigastric discomfort while seated. The symptoms aggravated upon bending forward. The patient had a BMI of 28 kg/m^2^. After dubious chest X-ray findings, computed tomography (CAT) evaluation showed an “upside-down stomach” situated in the thorax with an organoaxial rotation, along with a 9 × 11 cm hiatal defect. Endoscopy showed LA-B erosive esophagitis and confirmed the nonanatomical location of the gastroesophageal junction. The patient underwent a Belsey Mark IV antirefluxplasty with hiatal reconstruction through left posterolateral thoracotomy at a thoracic surgery institute. On the 6th postoperative day, the patient developed severe dysphagia. Repeat CAT imaging confirmed paraesophageal reherniation of the antrum (Figure 2), and the patient was referred to our institute for laparoscopic reoperative intervention.

Based on CAT scan findings, there was a high suspicion that the standard redo fundoplication with primary hiatal closure will be insufficient, and reinforcement of the crural closure will be necessary. In view of the debatable role of synthetic mesh reinforcement, our department evaluated the possibility of autologous dermis graft implementation, as we have utilized this graft in parastomal hernias. There is reemerging evidence in Hungarian as well as international literature for the clinical use in complicated incisional hernias [4–6], this experience has supported the effectivity of autologous dermis graft as reinforcement. After board review, the procedure protocol was approved by the Regional Research Ethics Committee. Following discussion with the patient and thorough explanation of the medical procedure, informed consent was obtained for dermis graft harvesting and implantation.

**Figure 3:**
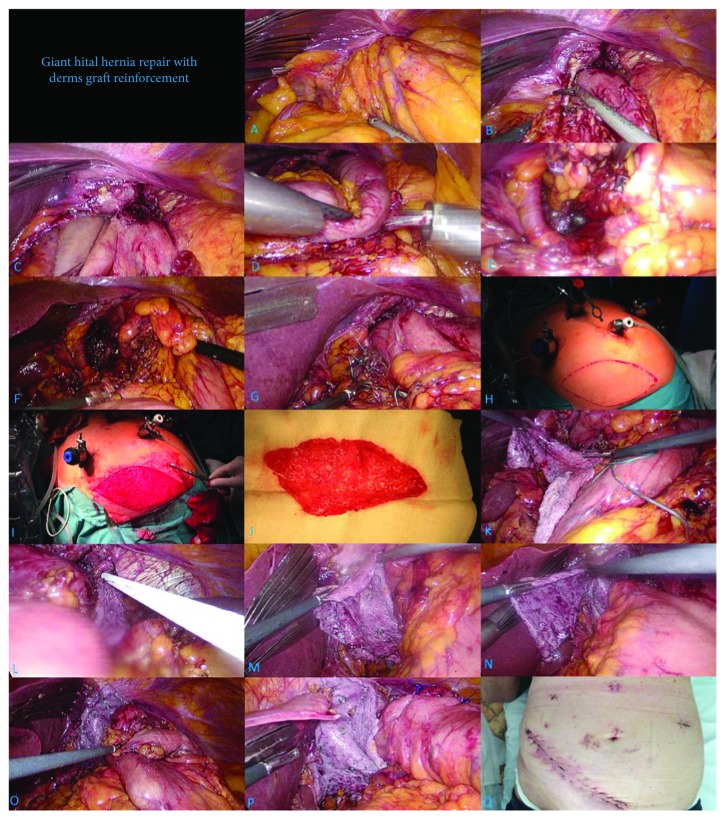
Surgical steps for giant hiatal hernia repair with dermis graft reinforcement: (a) initial intraoperative finding during redo surgery, 60% of the stomach is in the thoracic cavity; (b) adhesiolysis; (c) retraction of the mobile lesser curvature into the abdominal cavity; (d) stomach in the intra-abdominal position; (e) visualization of the crural stitch failure; (f) Belsey rear suture line left in place, fundoplication adequately mobilized; (g) suture repair of the defect; (h) harvesting site for dermis graft; (i) deepithelized skin flap; (j) dermis graft free of adipose tissue; (k) graft introduced into the abdomen; (l) graft fixation to the left diaphragmatic crura; (m) graft fixation to the right diaphragmatic crura; (n) excess graft removed; (o) anterior Dor's fundoplication; (p) final position of fundoplication wrap and the reinforced hiatal repair; (q) scar at 1-month follow-up.

The laparoscopic exploration was done on the 12th postoperative day. There was failure of the diaphragmatic crural stitches along with herniation of the antrum, pylorus, and greater omentum into the thorax (Figure 3(a)). We removed the second line of Belsey reconstruction that fixed the fundoplication to the left diaphragmatic crus. The lower esophageal sphincter (LES) was located in an intra-abdominal position, and the esophagus required no more mediastinal mobilization. After mobilization of the greater curvature, the hiatal defect was closed using 3 stitches of 0 braided nonabsorbable suture. Suture line seemed to be under tension, requiring reinforcement. Loose skin of the left lower abdomen was excised, thoroughly deepithelized, and cleaned from fatty tissue. The graft was rolled up and introduced into the abdomen through a 10 mm trocar. The graft was placed covering the suture line, also surrounding the esophagus in a U-shaped form, and was fixed to the diaphragm using titanium staples (Figures 3(l) and 3(m)), essentially resulting in a tensile repair with an onlay allograft reinforcement. The antireflux operation was completed creating an anterior (Dor) fundoplication around the distal esophagus. After desufflation, the abdominal skin defect was primarily closed with a running suture line. The total operative time was 195 minutes, including the preparation of the dermis graft which took 27 minutes.

## 3. Results

**Figure 4:**
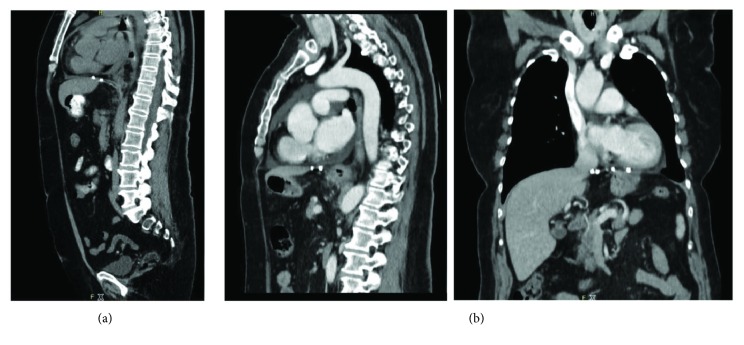
(a) CAT scan at 1-month follow-up shows anatomical position of abdominal viscera. (b) CAT scan at 7-month follow-up: sagittal and coronal reconstruction.

After an uneventful postoperative period, the patient was discharged on a pasty diet on the 3rd postoperative day. The patient was asymptomatic and doing well at the 14-day and 1-month follow-ups. The thoracoabdominal CAT scan at one-month follow-up showed normal anatomical position of the abdominal viscera (Figure 4(a)). Esophageal high-resolution manometry (HRM) revealed satisfactory LES function with adequate esophageal motility. The patient did not report any epigastric discomfort, and the self-perceived physical fitness was improved. Further follow-up was conducted 7 months postoperatively. Gastroduodenoscopy showed no signs of reflux, reherniation, or failure of fundoplication. HRM validated the continuous low-pressure zone at LES position. Repeat CAT scan at 7 months reassured normal anatomical position of viscera (Figure 4(b)). The patient was free of any abdominal or chest complaints. At 12-month telephone follow-up, the patient had no GI complaints and no respiratory complaints on exertion. There were minimal additional costs only due to the relatively longer operative time. Additional costs were significantly lower than the cost of available biological matrices.

## 4. Discussion

There is a building evidence favoring the need for reinforcement in the repair of giant hiatal hernias. The failure rate following the repair of large hiatal hernias without further reinforcement of the reconstructed diaphragm is unacceptably high [7]. Besides consistent esophageal and ventricular mobilization, a number of special interventions have been readily published to reduce recurrence rate. If the short esophagus is encountered intraoperatively, creating a Collis gastroplasty is justified. This has been reported to reduce the rate of recurrence [8]. As in the inguinal hernia repair, the mesh reinforcement was one of the earliest techniques to improve outcomes following a hiatal hernia repair. However, it carries a small but potentially fatal risk of complications in the long run. Mesh migration is one of these recognized complications and can further lead to stricture, perforation, or fistula formation of the esophagus or the wrap [9]. While the use of synthetic mesh prosthesis could lower the recurrence rate to an acceptable 12% [9], potentially severe complications cast a shadow on the generalized use of synthetic prosthesis, as life-threatening complications are far worse tolerated in the attendance of a benign disease [10–17]. After publication of these complications associated with the synthetic mesh graft, a notable number of novel approaches have emerged using various biological implants for reinforcement [18].

Bell et al. proposed an acellular dermal matrix allograft (with human dermal collagen derivative) reinforcement of the sutured hiatus using suture fixation, with results that encouraged further research [19]. The newer biological prostheses, however, further augment the cost of the hiatal repair [20]. Sasse et al. reduced the cost of reinforced hiatal hernia repair by using urinary bladder matrix for reinforcement, but the additional graft cost was still over 1000 USD/case [21]. Bjelovic et al. utilized autologous fascia lata graft for the reinforcement of the sutured diaphragmatic crura. They reported no recurrence in their 10 case series [22].

Autologous dermal grafts were one of the earliest biological matrices used in hernia repair in the early 20th century [23]. While there were numerous attempts of whole skin utilization as well [24], it was clarified in animal research that the complete and thorough deepithelization of the implanted graft not only facilitates incorporation but also mitigates infectious complications [25]. Shaffer presented a series of 27 massive inguinal and ventral hernia cases with dermal graft augmentation in 1956, where he also gave anecdotal mention of dermal graft use in a paraesophageal hernia case [26]. With the advancement in implantable synthetic material technology, the use of dermal grafts disappeared from international literature for the latter half of the 20th century. The use of autologous dermal graft in ventral and incisional hernias has reemerged in the last ten years, as the rates of recurrence and morbidity in complicated hernias remain high despite the use of synthetic meshes, and the use of biological matrices heavily augments procedural costs. Deepithelized dermal autograft has been used with promising results in these intricate cases [4–6]. As aforementioned literature presents, the implantation of dermal grafts into the structures of the abdominal wall can be considered safe.

Despite rapidly growing literature, there is still no gold standard for the type of graft to be used for reinforcement, in order to achieve the lowest rates of recurrence, complications, and procedural costs in giant hiatal hernias [10]. We present a novel approach to the reinforcement of hiatal hernia repair using a biological graft implant, with the benefits of allograft utilization. The increase in overall costs with our approach is only due to the increased operative time and consequent anesthetic costs. The method seems to have only advantages: graft is autologous with no risk of rejection; dermis is soft and pliable without risk of usuration or fistula formation of the esophageal wall; and there is no risk of severe visceral adhesions. The operative procedure is easily applicable and reproducible and requires routine laparoscopic instruments only. Numerous methods of deepithelization are utilized in regular plastic surgical work; one example is part of Schwarzmann breast reduction; the method applied should not affect the procedure as long as results is an even surfaced, completely epithelium, and fatty tissue-free graft.

The only mentionable disadvantage is the scar formation at the harvesting site. This can be minimized with a proper suturing and wound care. If patient composition permits, the optimal harvesting site is the loose skin of the lower abdomen. However, some patients with giant hiatal hernia often experience difficulty of oral intake resulting in a decreased BMI; in these cases, consultation with a plastic surgeon is recommended.

While comparing the additional burden of an abdominal skin scar to high-cost alternative grafts might be troublesome, the biological graft alternatives [20, 21] are not covered by the Hungarian State Insurance and therefore could be utilized only if purchased by the patient, and that is their major limiting factor in a number of socialized medical systems. The dermis graft thus has the advantage of being a viable, low-cost, and acceptable alternative to these patients. There is an ethical dilemma that lies within comparing the possible severe esophageal complications of nonbiological grafts, the high cost of biological implantables, and the scar formation of dermis graft harvesting. As all these three present in very different modalities, it should be entrusted to the thoroughly informed and enlightened patient to choose from the options available.

Our department has utilized the autologous dermis graft in primal reconstruction of giant hiatal hernias in three more cases which reconfirm the excellent short-term results; data collection for prospective case series is in progress.

## 5. Conclusions

We suggest onlay dermis graft reinforcement of the sutured diaphragmatic repair as a viable alternative in giant hiatal hernia repair. The method is eligible for further clinical evaluation. Further patient recruitment and long-term follow-up would be beneficial to determine the place of dermis graft reinforcement among the use of other biological matrices.

## Data Availability

Data are available through the corresponding author.

## Abbreviations

CAT: Computed tomography
GERD: Gastroesophageal reflux disease
HRM: High-resolution manometry
LES: Lower esophageal sphincter.
